# Impact of metastatic pattern and histologic subtype on PD-(L)1 inhibitor efficacy in HER2-negative advanced gastric and gastroesophageal cancer: a meta-analysis

**DOI:** 10.3389/fonc.2026.1857990

**Published:** 2026-06-29

**Authors:** Derek Tai, Kyung-il Kim, Pranati Shah, Daniel Park, Lucas Kim, Jianan Li, Claire Jung, Sofia Guzman, Gagandeep Brar, Shengyang Wu, Dani Castillo

**Affiliations:** 1 Department of Internal Medicine, Loma Linda University Medical Center, Loma Linda, CA, United States; 2 Department of Internal Medicine, Kaiser Permanente Fontana Medical Center, Fontana, CA, United States; 3 Department of Hematology and Oncology, Harbor-University of California, Los Angeles (UCLA) Medical Center, Torrance, CA, United States; 4 School of Medicine, Loma Linda University, Loma Linda, CA, United States; 5 Faculty of Kinesiology & Physical Education, University of Toronto, Toronto, ON, Canada; 6 Flintridge Preparatory School, La Cañada Flintridge, CA, United States; 7 Department of Medical Oncology and Therapeutics Research, City of Hope, Duarte, CA, United States; 8 Radiation Oncology/Gamma Knife, Valley/Mt Sinai Comprehensive Cancer Center, Paramus, NJ, United States

**Keywords:** gastric cancer, gastroesophageal junction, immunotherapy, Lauren classification, metastatic site, PD-1 blockade

## Abstract

**Background:**

The therapeutic benefit of PD-(L)1 blockade in advanced gastric and gastroesophageal junction adenocarcinoma (GC/GEJ) may vary by niche and tumor biology. We conducted a meta-analysis to evaluate how metastatic site and Lauren histologic subtype influence survival outcomes in HER2-negative GC/GEJ treated with PD-(L)1 inhibitor-based chemoimmunotherapy.

**Methods:**

A systematic PubMed, MEDLINE, and Embase search identified phase III randomized controlled trials published between 2021 and 2025 comparing IO plus platinum–fluoropyrimidine chemotherapy vs. chemotherapy alone in HER2-negative metastatic or unresectable GC/GEJ. Hazard ratios (HRs) for overall survival (OS) were pooled using random-effects models in the intention-to-treat (ITT) population and prespecified subgroups, including liver metastases (LM), peritoneal metastases (PM), Lauren histologic subtype, and PD-L1 expression assessed by combined positive score (CPS) or tumor area positivity score (TAPS). Between-study heterogeneity was assessed using the I² statistic, and analyses were conducted in R.

**Results:**

Six global trials including 5, 410 patients (CHECKMATE 649, ATTRACTION-4, KEYNOTE-859, ORIENT-16, RATIONALE-305, AND GEMSTONE-303) were analyzed. Overall, IO plus CT significantly improved OS compared with CT alone (HR 0.79, 95% CI 0.75-0.84; p<0.001). Survival benefit was observed in patients with and without LM (HR 0.75 and 0.82), whereas patients with PM derived limited benefit (HR 0.93). Intestinal-type tumors achieved greater OS improvement than diffuse-type tumors (HR 0.78 vs 0.87). PD-L1 CPS ≥5 predicted superior OS benefit (HR 0.70). Grade ≥3 adverse events were infrequent and hematologic.

**Conclusion:**

First-line chemoimmunotherapy prolongs survival in HER2-negative GC/GEJ. Reduced benefit in diffuse-type and peritoneal disease underscores heterogeneity and supports site- and histology-specific strategies.

**Systematic Review Registration:**

https://www.crd.york.ac.uk/PROSPERO/, identifier CRD420251174893.

## 1 Introduction

Gastric cancer (GC) ranks as the fifth most common malignancy and the fourth leading cause of cancer-related deaths worldwide with over 1, 000, 000 new cases and approximately 770, 000 deaths annually (1, 2). Despite advances in both diagnosis and systemic therapies, the prognosis of advanced or metastatic GC remains poor, with median overall survival (OS) of one year for patients receiving palliative chemotherapy (2–4). The development of immune checkpoint inhibitors, particularly antibodies targeting programmed cell death protein-1 (PD-1) or programmed cell death protein-ligand 1 (PD-L1), has improved the therapeutic landscape of gastrointestinal malignancies, including GC (5).

For patients with HER2-negative advanced gastric and gastroesophageal junction cancer (GC/GEJ), the use of PD-(L)1 inhibitors in combination with first-line chemotherapy has markedly improved clinical outcomes compared with chemotherapy alone (4, 6–11). The addition of PD-(L)1 blockade to platinum-fluoropyrimidine chemotherapy has improved OS and progression free survival (PFS) in multiple phase III trials, with the greatest and most consistent benefit observed in patients with PD-L1 combined positive score (CPS) ≥ 5, establishing chemo-immunotherapy as the preferred first-line approach in this population (4, 6–10). Despite these advances, therapeutic benefits remain mixed with overall response rates of 40%-50% (12), reflecting the complex interplay between tumor biology, host immunity, and the tumor microenvironment (TME) that influences patient response to immune checkpoint inhibitors.

Two key elements of immune responsiveness in GC are the metastatic site and Lauren histologic subtype. Peritoneal metastases (PM) occur in up to 40% of GC/GEJ and are associated with an immune-suppressive TME characterized by low PD-L1 expression, sparse tumor-infiltrating lymphocytes, and immunosuppressive cytokine signaling (13–15). In contrast, liver metastases (LM) represent another immunologically distinct pattern in which high densities of myeloid-derived suppressor cells and tumor-associated macrophages create a tolerogenic environment rich in TGF-β and IL-10, blunting effector T-cell infiltration and diminishing checkpoint blockade (16, 17). The Lauren classification, which divides GC into intestinal and diffuse types, provides additional biologic and immunologic insight (18). Intestinal-type GC exhibits an immune-inflamed TME with high PD-L1 expression, abundant CD8^+^ T-cell infiltration, and upregulated interferon-γ signaling *in vivo*, while diffuse-type GC shows an immune-excluded phenotype marked by dense desmoplastic stroma, TGF-β–driven cancer-associated fibroblast (CAF) activation and limited immune cell penetration (19–21).

Several phase III trials have evaluated outcomes by PD-L1 status, including CHECKMATE 649, KEYNOTE-859, ORIENT-16, and ATTRACTION-4; however, the combined impact of tumor histology and metastatic pattern on immunotherapy efficacy in advanced GC/GEJ remains unclear (4, 6–10). As PD-(L)1 inhibitors become a standard-of-care option for patients with HER2-negative GC/GEJ cancers, identifying biological and anatomical determinants of response is essential for optimal patient selection for combination immune checkpoint inhibitor and chemotherapy. To address this, we conducted a meta-analysis of six phase III randomized controlled trials evaluating immune checkpoint inhibitor combined with systemic chemotherapy in patients with advanced HER2-negative GC/GEJ.

## 2 Methods

### 2.1 Search strategy and study selection

This systematic review and meta-analysis adhered to PRISMA 2020 guidelines and was registered in PROSPERO (https://www.crd.york.ac.uk/PROSPERO/view/CRD420251174893)). A comprehensive literature search was conducted using PubMed, MEDLINE, and Embase for studies published between January 1, 2021, and September 30, 2025. Manual screening of reference lists from eligible studies and relevant review articles was additionally performed to identify potentially missed studies. Search terms included “gastric cancer”, “stomach neoplasm”, “gastroesophageal junction cancer”, “PD-1 inhibitor”, “PD-L1 inhibitor”, “nivolumab”, “pembrolizumab”, “tislelizumab”, “sintilimab”, “sugemalimab”, “chemotherapy”, “HER2-negative”, and/or “HER2 negative”.

Only phase III randomized controlled trials comparing PD-(L)1 inhibitor plus chemotherapy vs. chemotherapy plus placebo or chemotherapy alone in HER2-negative advanced GC/GEJ published in English were included. Two independent reviewers DT and KK screened all titles, abstracts, and full texts. Discrepancies in study selection and inclusion criteria were resolved by consensus or third reviewer DC. Conference abstracts, case reports, and non–phase III trials were excluded.

### 2.2 Data extraction and outcomes

Two reviewers independently extracted relevant data from eligible trials, including trial name, NCT number, year of trial publication, PD-(L)1 agent, sample size, line of therapy, and chemotherapy regimen. Patient characteristics such as PD-L1 expression threshold, histologic subtype, and metastatic distribution were also assessed. Finally, OS hazard ratios (HRs) for subgroups such as presence of LM, presence of PM, Lauren histologic subtype (intestinal or diffuse), and PD-L1 expression status were evaluated. The primary outcome was OS in predefined subgroups. Secondary outcomes comprised incidence of grade ≥3 treatment-related adverse events.

### 2.3 Statistical analysis

All statistical analyses were performed using R (version 4.3.2) with the meta and metafor packages. Log-transformed HRs and 95% confidence intervals (CIs) were extracted directly or derived from published Kaplan-Meier estimates. Pooled HRs were calculated using a random-effects model to account for between-study variability. An HR < 1 indicated a survival benefit with PD-(L)1 inhibitor and chemotherapy vs. chemotherapy alone. Study heterogeneity between studies was quantified using the *I²* statistic, with values of 25%, 50%, and 75% representing low, moderate, and high heterogeneity, respectively. *I²* estimates may be biased when the number of included studies is small, therefore, we additionally examined the magnitude and direction of individual study effects and confirmed the consistency of findings using a random-effects model and sensitivity analyses. Forest plots were generated to display effect sizes across subgroups. Assessment of publication bias was performed using funnel plot analysis. For each included study, the logarithm of the hazard ratio (log[HR]) was plotted against its corresponding standard error. The pooled effect estimate was indicated by a vertical reference line, and pseudo-95% confidence limits were overlaid to aid visual interpretation. Visual symmetry of studies around the pooled estimate was interpreted as suggesting a low risk of publication bias, whereas asymmetry was considered suggestive of potential small-study effects or between-study heterogeneity.

## 3 Results

### 3.1 Study selection and characteristics

**Figure 1:**
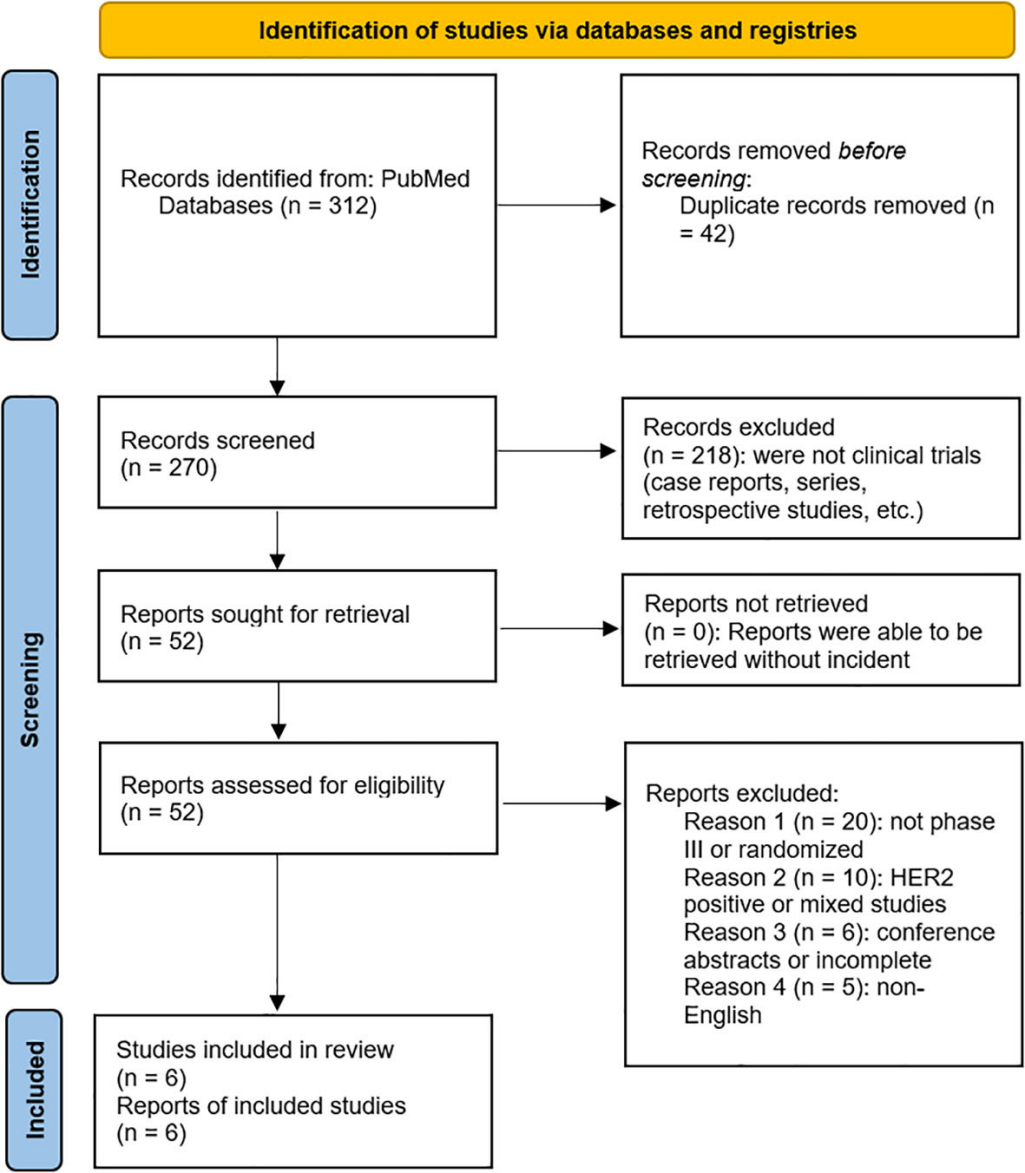
PRISMA 2020 flowchart. Flow diagram illustrating identification, screening, eligibility assessment, and final inclusion of phase III randomized trials evaluating first-line PD-(L)1 inhibitor plus chemotherapy in HER2-negative advanced GC/GEJ.

A literature search produced 312 records from PubMed, MEDLINE, and Embase. After screening, six phase III randomized controlled trials met the eligibility criteria, including a total of 5, 410 patients with HER2-negative advanced GC/GEJ treated with first-line therapy of CT with or without a PD-(L)1 inhibitor. A PRISMA 2020 Flowchart was completed to illustrate the selection process. (**Figure 1**). Chemotherapy regimens comprised oxaliplatin or cisplatin combined with fluoropyrimidines such as 5-FU, S-1, or capecitabine. PD-(L)1 inhibitors included nivolumab, pembrolizumab, sintilimab, tislelizumab, and sugemalimab. PD-L1 positivity was defined as CPS ≥5 (or tumor area positivity [TAP] ≥5% in RATIONALE-305) to align with the primary efficacy populations and prespecified subgroup analyses reported in the included phase III trials. Although clinical guidelines may support PD-(L)1 inhibitor use in patients with CPS ≥1, the CPS ≥5 threshold was selected to maximize methodological consistency across studies and because this subgroup demonstrated the most reproducible survival benefit in pivotal registration trials. In addition to CPS, RATIONALE-305 used TAP ≥ 5% to define PD-L1 positive patients. Median follow-up durations ranged from 16 to 31 months.

Key characteristics were described for all included trials (**Table 1**). The trials analyzed were: GEMSTONE-303 (sugemalimab plus chemotherapy [capecitabine and oxaliplatin] vs. chemotherapy plus placebo) (9), RATIONALE-305 (tislelizumab plus chemotherapy [capecitabine and oxaliplatin or cisplatin and 5-fluorouracil] vs. chemotherapy plus placebo (8), KEYNOTE-859 (pembrolizumab plus chemotherapy [fluorouracil and cisplatin or capecitabine and oxaliplatin] vs. chemotherapy plus placebo) (6), ORIENT-16 (sintilimab plus chemotherapy [capecitabine and oxaliplatin] vs. chemotherapy plus placebo) (7), ATTRACTION-4 (nivolumab plus chemotherapy [oxaliplatin and S-1 or oxaliplatin and capecitabine] vs. chemotherapy plus placebo) (10), CHECKMATE-649 (nivolumab plus chemotherapy [capecitabine and oxaliplatin or leucovorin, fluorouracil, and oxaliplatin] vs. chemotherapy alone) (4, 11).

**Table 1:** Baseline characteristics and regional distribution of the six pivotal phase III trials in advanced GC/GEJ.

Study	Year of Publication	ITT Population, n (total, n)	Treatment group	Control group	Region(s)
GEMSTONE-303 (NCT 03802591)	2025	241 (479)	Sugemalimab + chemotherapy (capecitabine and oxaliplatin)	Placebo + chemotherapy	Asia
RATIONALE-305 (NCT 03777657)	2024	503 (997)	Tislelizumab + chemotherapy (capecitabine and oxaliplatin or cisplatin and 5-fluorouracil)	Placebo + chemotherapy	Asia, Europe, North America
KEYNOTE-859 (NCT 03675737)	2025	790 (1579)	Pembrolizumab + chemotherapy (fluorouracil and cisplatin or capecitabine and oxaliplatin)	Placebo + chemotherapy	Asia, North America, Australia
ORIENT-16 (NCT 03745170)	2024	327 (650)	Sintilimab + chemotherapy (capecitabine and oxaliplatin)	Placebo + chemotherapy	Asia
ATTRACTION-4 (NCT 04370410)	2022	362 (724)	Nivolumab + chemotherapy (oxaliplatin and S-1 or oxaliplatin and capecitabine)	Placebo + chemotherapy	Asia
CHECKMATE 649 (NCT 02872116)	2021	789 (1581)	Nivolumab + chemotherapy (capecitabine and oxaliplatin or leucovorin, fluorouracil, and oxaliplatin)	chemotherapy alone	Asia, Europe, North America

ITT, intent-to-treat; NCT, National Clinical Trial.

### 3.2 Overall survival

**Figure 2:**
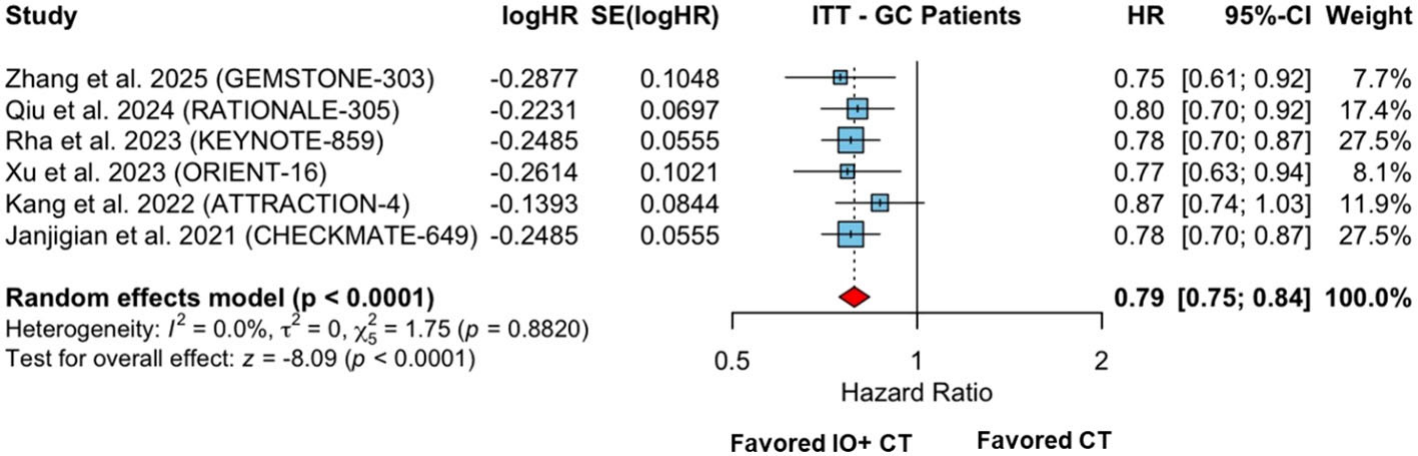
Forest plot analysis of overall survival in all GC/GEJ patients. Pooled hazard ratios for overall survival comparing PD-**(L)**1 inhibitor plus chemotherapy versus chemotherapy alone in HER2-negative advanced GC/GEJ.

Forest plot shows HRs for OS comparing immune checkpoint inhibitor plus platinum–fluoropyrimidine chemotherapy vs. chemotherapy alone across randomized phase III trials in the ITT population. Chemo-immunotherapy was associated with a significant improvement in OS (pooled HR 0.79, 95% CI 0.75–0.84; p < 0.0001) with no detectable between-study heterogeneity demonstrating a consistent treatment effect across studies (**Figure 2**). PFS subgroup data were inconsistently reported across trials and therefore were not included in pooled analyses.

### 3.3 Subgroup analyses by metastatic site

**Figure 3:**
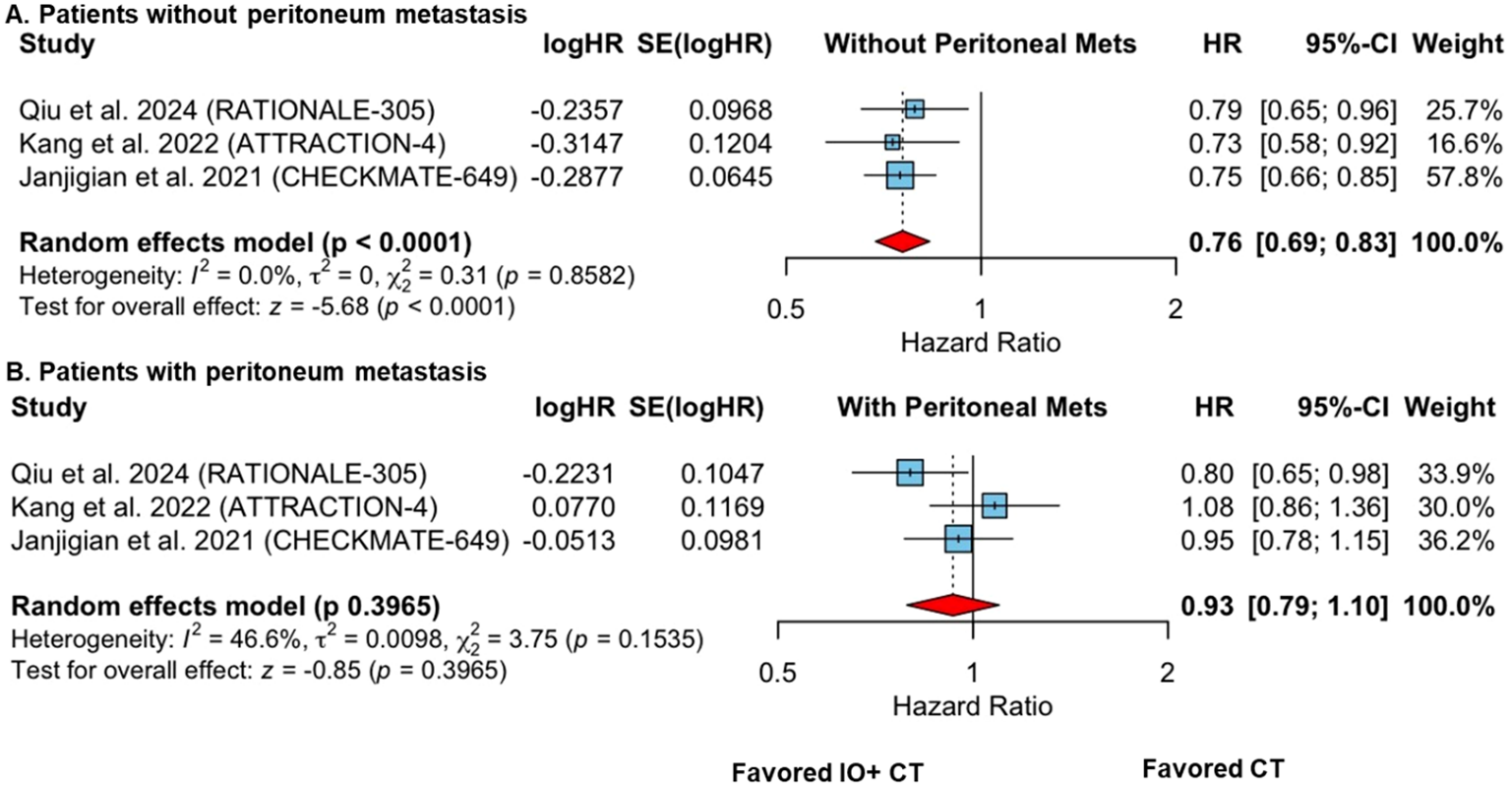
Forest plot analysis of overall survival in GC/GEJ patients without peritoneal metastasis and with peritoneal metastasis. Pooled hazard ratios for overall survival stratified by the **(A)** presence or **(B)** absence of peritoneal metastases.

**Figure 4:**
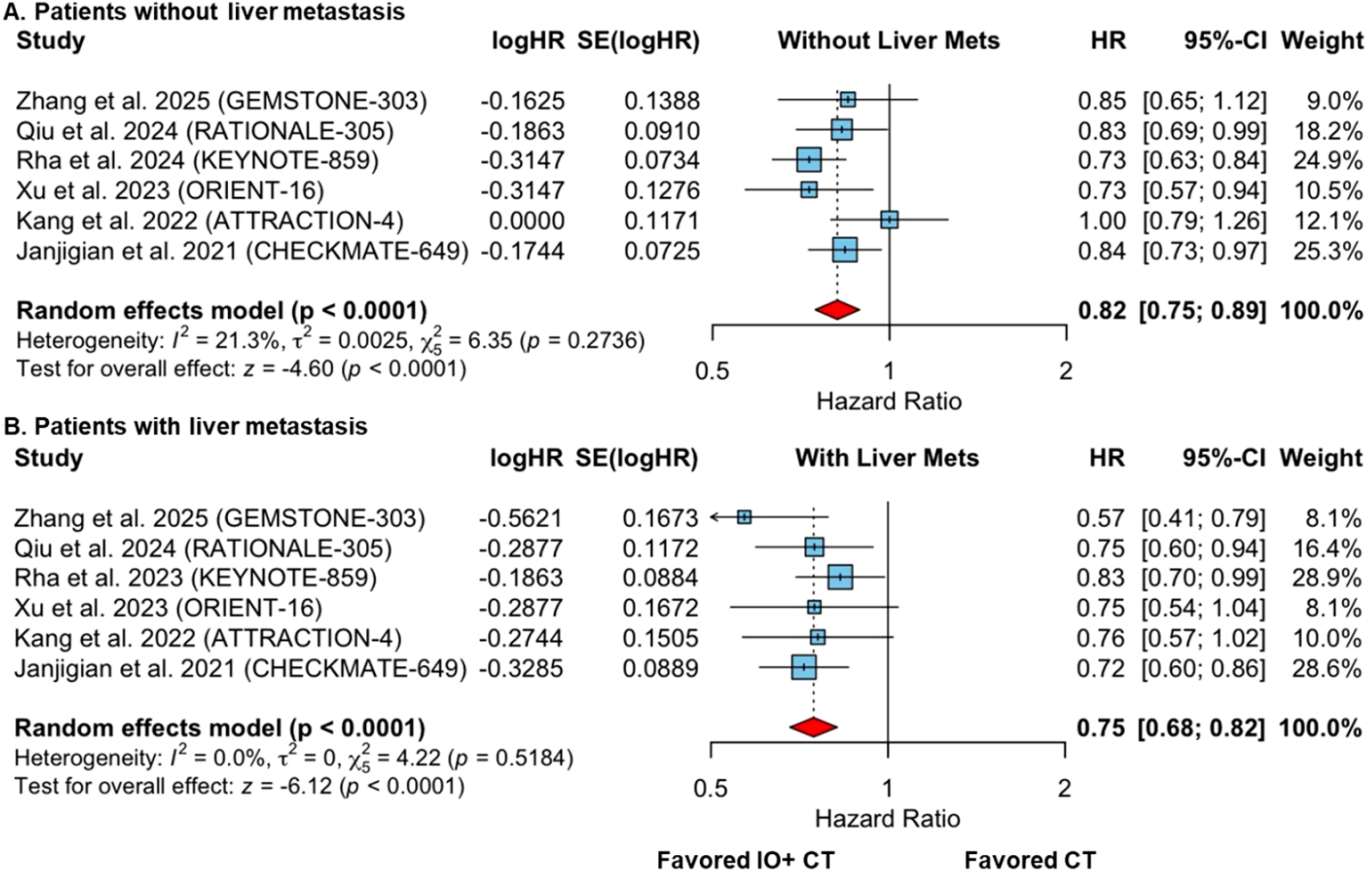
Forest plot analysis of overall survival in GC patients without liver metastasis and with liver metastasis. Pooled hazard ratios for overall survival stratified by the **(A)** presence or **(B)** absence of liver metastases.

Among GC/GEJ cancer patients without PM, the addition of immune checkpoint inhibitor to platinum-fluoropyrimidine chemotherapy was associated with a significant improvement in OS compared with chemotherapy alone (pooled HR 0.76, 95% CI 0.69–0.83; p < 0.0001), with no evidence of between-study heterogeneity (*I²* = 0%). In contrast, among patients with PM, no statistically significant OS benefit was observed with immune checkpoint inhibitor and chemotherapy (pooled HR 0.93, 95% CI 0.79–1.10; p = 0.40), and moderate heterogeneity was present (*I²* = 46.6%) (**Figure 3**). In contrast, among patients with LM, PD-(L)1 inhibitor plus chemotherapy significantly prolonged OS (HR = 0.75; 95% CI 0.68–0.82; p < 0.001; *I²* = 10.5%), with a similar benefit observed in patients without LM (HR = 0.82; 95% CI 0.75–0.89; p < 0.001; *I²* = 0%) (**Figure 4**). Patients categorized as negative for LM or PM frequently had alternative sites of metastatic dissemination, including lymph node, lung, bone, or distant nonperitoneal soft tissue involvement, as defined within the original trial populations. Since subgroup analyses were based on the presence or absence of specific metastatic sites rather than mutually exclusive metastatic categories, patients may have harbored additional concurrent metastatic lesions.

### 3.4 Histological and biomarker subgroups

When stratified by Lauren histologic subtype, patients with intestinal-type GC/GEJ cancer derived a significant OS benefit from immune checkpoint inhibitor plus chemotherapy compared with chemotherapy alone (pooled HR 0.78, 95% CI 0.69–0.88; p < 0.0001), with no evidence of between-study heterogeneity (*I²* = 0%). In contrast, among patients with diffuse-type disease, the survival benefit was attenuated and did not reach statistical significance (pooled HR 0.87, 95% CI 0.74–1.01; p = 0.067), with moderate heterogeneity observed across trials (*I²* = 44.3%). These findings indicate that histologic subtype influences the magnitude of benefit from first-line chemoimmunotherapy, with intestinal-type tumors demonstrating more consistent and clinically meaningful responses than diffuse-type tumors. (**Supplementary Figure S1**).

Across studies reporting PD-L1 status, patients with CPS ≥5 or TAP ≥5% derived OS benefit (HR = 0.70; 95% CI, 0.64–0.77; p < 0.0001; *I²* = 0%) while PD-L1-negative (CPS <5) subgroup data were insufficient for pooling. Forest plots depict HRs for OS comparing immune checkpoint inhibitor plus platinum-fluoropyrimidine chemotherapy vs. chemotherapy alone across randomized phase III trials. In the overall PD-L1–positive population, immune checkpoint inhibitor plus chemotherapy was associated with a significant survival benefit (pooled HR 0.70, 95% CI 0.64–0.77; p < 0.0001) with no detectable heterogeneity. Among PD-L1-positive patients with LM, a consistent and pronounced OS benefit was observed (pooled HR 0.65, 95% CI 0.56–0.75; p < 0.0001). In contrast, no significant survival benefit was demonstrated in PD-L1-positive patients with PM (pooled HR 0.93, 95% CI 0.79–1.10; p = 0.40), with moderate between-study heterogeneity. Random-effects models were applied throughout. (**Supplementary Figure S2****).**

### 3.5 Safety

Grade ≥3 treatment-related adverse events occurred more frequently with PD-1 inhibitors plus chemotherapy compared to chemotherapy alone, primarily hematologic toxicities such as anemia, neutropenia, and thrombocytopenia. Immune-related adverse events were uncommon and mostly grade 1-2; grade ≥3 immune-related adverse events occurred in 4-8% of patients. Treatment discontinuation and treatment-related mortality were < 2% in both treatment arms and comparable in all trials.

## 4 Discussion

A recent pooled meta-analysis of 163 studies across multiple tumor types demonstrated that LM were associated with inferior PFS among patients treated with immune checkpoint inhibitor (22). These findings underscore the heterogeneity of immunotherapy efficacy across metastatic sites and tumor types, providing the rationale for the present meta-analysis focused on HER2-negative advanced GC/GEJ cancers. Our pooled analysis demonstrated that first-line chemoimmunotherapy consistently improved OS in patients with advanced HER2-negative GC/GEJ, regardless of LM status and across intestinal-type tumors. In contrast, the attenuated benefit observed in diffuse-type tumors and those with PM suggests a more immunosuppressive TME that may limit immune checkpoint inhibitor efficacy. Conversely, the greater survival advantage seen in intestinal-type and LM subgroups likely reflects a more immune-inflamed phenotype with enhanced reliance on checkpoint signaling pathways.

### 4.1 Metastatic site as a determinant of immunotherapy efficacy

PM may represent a biomarker of relative PD-1/PD-L1 resistance, highlighting the need for intensified or alternative approaches such as anti-TGF-β-based combinations or intraperitoneal immunotherapy (23). Among patients with LM, the addition of immune checkpoint inhibitor to chemotherapy significantly prolonged OS with a comparable benefit also observed in those without LM. Interestingly, the magnitude of benefit appeared slightly greater in the LM subgroup, suggesting that immune checkpoint inhibitor-based combination therapy remains effective even in patients traditionally considered less responsive to immunotherapy. These findings challenge the prior notion that hepatic metastases confer intrinsic resistance to PD-(L)1 blockade and underscore that immune checkpoint inhibitor can still provide meaningful clinical benefit in this population. These findings challenge the prevailing assumption that liver metastases universally confer resistance to immune checkpoint inhibition in GC/GEJ. The low heterogeneity between each study reinforces the reproducibility of this effect and suggests that combination therapy may overcome local immunosuppression within hepatic lesions through enhanced antigen presentation, increased CD8+ T-cell priming, or systemic cytokine modulation induced by chemotherapy (24, 25). In contrast, patients with PM did not experience an OS benefit as seen by a pooled HR of 0.93 and moderate heterogeneity. This lack of efficacy likely reflects the unique immune-excluded biology of the peritoneal TME (26, 27).

From a clinical perspective, patients with PM represent a high-risk subgroup that may require treatment strategies beyond conventional systemic chemoimmunotherapy. For selected patients with limited peritoneal disease burden and preserved performance status, locoregional approaches including cytoreductive surgery with HIPEC or investigational PIPAC-based strategies may improve intraperitoneal drug exposure and disease control (3, 27–29). Emerging studies evaluating intraperitoneal chemotherapy combined with immune checkpoint inhibitors have supported evidence of immune remodeling within the peritoneal cavity and increased immune cell infiltration, supporting the feasibility of peritoneal-directed immunotherapy approaches (27, 30, 31). Additionally, combination regimens incorporating VEGF inhibition, TGF-β blockade, or stromal-targeting agents may help overcome the immune-excluded phenotype characteristic of PM (19–21, 23, 32). Given the limited survival benefit observed in our pooled analysis, studies evaluating peritoneal-specific therapeutic strategies may provide beneficial data in improving patient outcomes (13, 27, 33).

PM in GC is characterized by dense stromal fibrosis, low PD-L1 expression on both tumor and immune cells, poor vascularization, and limited T-cell infiltration (13–15).These factors limit both drug penetration and immune activation, leading to reduced immune checkpoint inhibitor sensitivity (26, 27, 34). PM in GC represents a distinct biological and pharmacological niche where the efficacy of systemic therapies is markedly reduced (33). Unlike hematogenous metastases, the peritoneal TME exhibits distinct anatomic and immunologic barriers that limit therapeutic efficacy (26, 35, 36). Structurally, it is characterized by hypoxia, aberrant vasculature, and upregulated VEGF signaling, which together lead to poor intratumoral drug penetration following systemic administration. Immunologically, the peritoneal TME displays dense stromal desmoplasia, enrichment of tumor-associated macrophages and regulatory T cells, and elevated levels of immunosuppressive cytokines such as TGF-β, IL-6, and VEGF, collectively fostering an immune-excluded, myeloid-dominant milieu that confers primary resistance to immune checkpoint inhibitor. Tumor-intrinsic mechanisms, such as epithelial-mesenchymal transition, CDH1 loss, and low tumor mutational burden, further diminish antigenicity and T-cell infiltration. Additionally, malignant ascites function as a barrier to drug penetration, impairing pharmacologic delivery to peritoneal tumor deposits (33). The ascitic microenvironment further promotes dissemination of multicellular tumor spheroids capable of evading both cytotoxic and immune-mediated clearance (26, 27, 34).

Anatomic and biological barriers inherent to PM underscore the limited efficacy of systemic chemo-immunotherapy in GC and provide a strong rationale for regionally delivered approaches, such as pressurized intraperitoneal aerosol chemotherapy (PIPAC) (3, 28), hyperthermic intraperitoneal chemotherapy (HIPEC) (27), and immunomodulatory combinations to overcome local drug delivery and immune evasion hurdles (29, 30, 34, 37). Future strategies may consider stroma-modulating agents, regional immune checkpoint inhibitor delivery, or combination approaches such as PD-(L)1 blockade with anti-TGF-β or VEGF inhibitors to overcome the stromal and vascular barriers that limit immune cell trafficking and cytotoxic activity in the peritoneal TME (19–21).

### 4.2 Distinct transcriptional and immune programs define the peritoneal vs. hepatic metastatic niches

The high prevalence of PM represents a major obstacle in GC/GEJ management (38). Unlike the downstream site of progression seen in LM in GC, PM in GC represents a distinct ecological niche with its own stromal, immune, and transcriptional programs (39). Single-cell and spatial profiling of GC peritoneal lesions have demonstrated that the peritoneal compartment is enriched for CAFs, including inflammatory CAFs (iCAFs) and myofibroblastic CAFs (myCAFs), as well as SPP1^+^/C1QC^+^ macrophage populations (32, 40). These stromal and myeloid cells cooperate to generate a TGF-β-rich, fibrosis-like TME that physically excludes effector T cells and limits productive antitumor immunity. In parallel, pathway-level comparisons between primary gastric tumors, PM, and LM show that PM is uniquely enriched for EMT signatures, stromal infiltration programs, and dendritic cell-CAF signaling circuits (27, 28, 34). In contrast, LM exhibit a more immune-permissive landscape, with reduced CAF density, higher infiltration of cytotoxic T and natural killer cells, and distinct metabolic and cytokine signaling profiles (16, 17). The hepatic niche is also shaped by resident Kupffer cells and liver sinusoidal endothelial cells, which modulate immune tolerance differently from the stromal fibrosis and ascitic milieu characteristic of PM (16, 17).

Immune suppression in the peritoneal cavity is layered. Regulatory B cells producing IL-10, tolerogenic macrophages, and stromal remodeling have been described in malignant ascites and nodules of PM from GC (41), suggesting a locally immunosuppressive setting that promotes tumor survival and immune escape. Fibroblast-myeloid crosstalk, particularly CAF-driven polarization of macrophages toward protumor phenotypes, appears central to this process (32). In PM samples from patients treated with intraperitoneal chemotherapy plus systemic immunotherapy, transcriptomic profiling revealed increased immune cell infiltration, activation of inflammatory pathways, and metabolic reprogramming of peritoneal adipose tissue features consistent with an immune-active TME within the metastatic fat-pad niche. These findings suggest that peritoneal dissemination can be immunologically remodeled, support developing peritoneal-specific strategies such as TGF-β blockade, CAF/myeloid targeting, and intraperitoneal immunotherapy (31).

### 4.3 Histological subtype and the tumor immune microenvironment

The observed divergence in response between intestinal-type and diffuse-type GC mirrors known immunogenomic differences. Intestinal-type tumors, which are more likely to exhibit microsatellite instability, Epstein-Barr virus positivity, or intermediate-to-high tumor mutational burden, typically express higher PD-L1 expression and greater infiltration of cytotoxic T cells (19, 20). These features create an immune-inflamed phenotype which is more amenable to immune checkpoint inhibitor.

Diffuse-type tumors, often associated with CDH1 mutations and stromal desmoplasia, exhibit a stromal-restricted immune microenvironment characterized by dense collagenous matrices and activation of the collagen-CD44 axis, which hinders immune cell infiltration and promotes T-cell dysfunction (19). Intestinal type GC typically exhibits an “immune-inflamed” phenotype with abundant CD8^+^ T cells, dendritic cells, and interferon-γ-related gene expression, which are predictive of checkpoint inhibitor response (19). By contrast, diffuse-type GC shows low immune infiltration, fewer antigen-presenting cells, limited cytokine signaling and reduced levels of CD8+ tumor-infiltrating lymphocytes circulating natural killer cells and regulatory T cells (42). As immune checkpoint inhibitor such as nivolumab, pembrolizumab, sintilimab, tislelizumab, and sugemalimab enter first-line options, histologic and molecular stratification will be key to personalizing treatment and combination strategies (4, 6–11).

Diffuse-type gastric cancer recurrence remains particularly challenging because of its tendency for peritoneal dissemination, infiltrative growth patterns, and stromal-rich microenvironment (18–21). Although first-line PD-(L)1 inhibitor-based chemoimmunotherapy remains a standard treatment option, the reduced benefit observed in diffuse-type tumors in our analysis suggests that additional therapeutic approaches may be necessary. Therapeutic strategies aimed at overcoming stromal exclusion and immune suppression, including anti-TGF-β approaches, cancer-associated fibroblast targeting, and combination immunotherapy regimens, may be particularly relevant in this biologically distinct subtype (19, 21, 32, 40). Additional research in biomarker-driven clinical trials investigating novel immunotherapy combinations and TME-targeting therapies may benefit future patients with diffuse-type gastric cancer given the persistently poor outcomes associated with current conventional treatments (18–21, 42).

### 4.4 PD-L1 expression and the interplay with tumor biology

The consistent benefit observed in PD-L1-positive patients across LM reinforces PD-L1 expression as a validated biomarker of response. However, data for PD-L1-negative patients were insufficient for pooled analysis, and potential benefit in this subgroup cannot be excluded. Furthermore, our findings suggest that PD-L1 expression alone may be insufficient to capture the complexity of the immune landscape in GC/GEJ. PD-1 inhibitors generally achieve broader checkpoint blockade and may provide greater efficacy in PD-L1-low or PD-L2-expressing tumors, while PD-L1 inhibitors may have distinct safety and myeloid-modulating effects. The observed differences in gastric/GEJ cancer likely reflect a combination of biologic scope (PD-L2 sparing), trial heterogeneity, and differential TME engagement. The interaction between PD-L1 expression, metastatic niche, and histological subtype potentially influences treatment outcomes in a multifactorial manner. For instance, PD-L1-positive diffuse tumors may resist therapy due to stromal barriers, while PD-L1-negative intestinal tumors may respond via an inflamed TME or Epstein-Barr virus-driven immunity.

## 5 Limitations

Several limitations should be acknowledged. First, only first-line, phase III randomized trials were analyzed, which may limit generalizability to later-line settings; however, this approach ensures inclusion of the highest-quality evidence with standardized treatment protocols and survival endpoints. Second, this meta-analysis relied on aggregate, study-level data, precluding patient-level adjustment for confounders such as performance status, tumor burden, or subsequent therapies. Nonetheless, trial-level stratification and sensitivity analyses help mitigate these effects and strengthen the internal validity of pooled results. Third, heterogeneity in PD-L1 assay platforms, scoring thresholds, and chemotherapy backbones across studies may introduce variability in subgroup outcomes, but this diversity also reflects real-world practice and enhances the external applicability of the findings. PFS subgroup analyses were not feasible because several included trials did not uniformly report site-specific or histology-specific PFS hazard ratios. Moreover, not all studies reported Lauren subtype or PM-specific HR, limiting the precision of pooled estimates; ongoing and future trials incorporating molecular and histologic annotations will help refine these insights. Subgroup analyses according to metastatic site were exploratory and based on aggregate trial-level data and since patients could harbor multiple concurrent metastatic sites, the independent contribution of individual metastatic niches to treatment response could not be fully isolated. Finally, newer immunotherapeutic and regional strategies such camrelizumab, toripalimab, and PIPAC were not included as they have not yet been evaluated in first-line phase III GC/GEJ trials (43). The treatment field will continue to evolve as new drugs and studies are investigated.

## 6 Future directions

Future research should focus on integrating spatial and single-cell immune profiling to elucidate mechanisms of resistance to immune checkpoint inhibition in peritoneal and diffuse-type disease. Evaluating mechanism-driven combination regimens, including PD-(L)1 inhibitors paired with anti-fibrotic, anti-VEGF, or anti-TGF-β agents, as well as locoregional therapies like HIPEC or PIPAC may help remodel immune-TME and enhance therapeutic response. Several of these strategies are currently under investigation in preclinical models or early-phase trials in GC, including intraperitoneal chemotherapy combined with immune checkpoint inhibitor (22–24, 27). In parallel, prospective, biomarker-driven clinical trials stratified by histologic subtype and metastatic pattern are warranted to validate subgroup-specific therapeutic strategies. Finally, the incorporation of immune gene expression signatures could enable real-time monitoring of treatment response and resistance evolution.

## 7 Conclusions

This meta-analysis shows that adding immune checkpoint inhibitor to chemotherapy significantly improves OS in HER2-negative advanced GC/GEJ. The magnitude of benefit varies by histology and metastatic site, with reduced efficacy in diffuse-type and PM but enhanced responsiveness in intestinal-type and LM. These patterns suggest distinct TME that influence treatment outcomes. Incorporating histologic and metastatic profiling into trial design and clinical decision-making will optimize patient selection. Future studies integrating spatial and molecular immune profiling are needed to guide biomarker-driven strategies and advance precision immunotherapy in GC/GEJ.

## Data Availability

The original contributions presented in the study are included in the article/**Supplementary Material**. Further inquiries can be directed to the corresponding author.

## Supplementary material

Supplementary Figure S1 **(A)** Pooled analysis of four randomized phase III trials assessing IO plus CT vs. CT plus placebo (or CT alone in Checkmate 649) in patients with advanced GC with intestinal subtype showed OS benefit with IO + CT (HR = 0.78; 95% CI, 0.69–0.87; p < 0.0001) with no observed heterogeneity (I^2^ = 0%). **(B)** Pooled analysis of four randomized phase III trials assessing IO plus CT vs. CT plus placebo (or CT alone in Checkmate 649) in patients with advanced GC with diffuse subtype showed significant OS benefit for IO + CT (HR = 0.87; 95% CI, 0.74–1.01; p = 0.0067) with moderate heterogeneity (I^2^ = 39%). Individual study estimates are represented by blue squares (size proportional to study weight), and the pooled effect is shown as a red diamond. Values < 1.0 indicate improved survival with IO plus CT. CI, confidence interval; CT, chemotherapy; HR, hazard ratio; IO, PD-(L)1 inhibitor; SE, standard error.

Supplementary Figure S2 OS benefit of first-line chemo-immunotherapy in PD-L1–positive gastric and gastroesophageal junction cancer, stratified by metastatic site. Forest plots depict HRs for OS comparing IO plus CT versus CT alone across randomized phase III trials. **(A)** In the overall PD-L1–positive population, chemo-immunotherapy was associated with a significant survival benefit (pooled HR 0.70, 95% CI 0.64–0.77; p < 0.0001) with no detectable heterogeneity. **(B)** Among PD-L1–positive patients with LM, a consistent and pronounced OS benefit was observed (pooled HR 0.65, 95% CI 0.56–0.75; p < 0.0001). **(C)** No significant survival benefit was demonstrated in PD-L1–positive patients with PM (pooled HR 0.93, 95% CI 0.79–1.10; p = 0.40), with moderate between-study heterogeneity. OS, overall survival; CT, chemotherapy; HR, hazard ratio; IO, PD-(L)1 inhibitor; PM, Peritoneum metastasis; LM, liver metastases; SE, standard error.
