# Supplementary figures and images for: Impact of metastatic pattern and histologic subtype on PD-(L)1 inhibitor efficacy in HER2-negative advanced gastric and gastroesophageal cancer: a meta-analysis
Source: Front Oncol. 2026 Jun 29;16:1857990. doi: 10.3389/fonc.2026.1857990 (PMC13357174; doi:10.3389/fonc.2026.1857990)

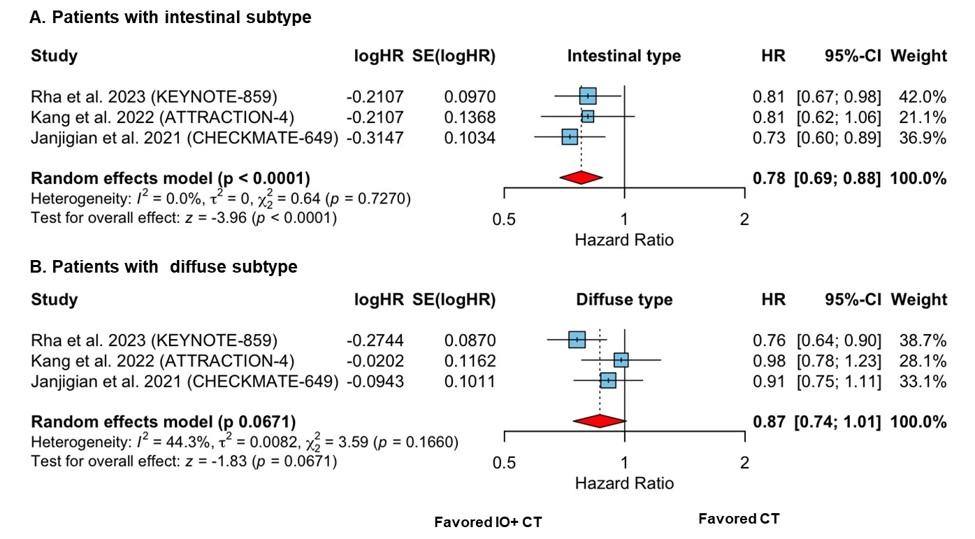

Supplement: Supplementary Figure S1 — (A) Pooled analysis of four randomized phase III trials assessing IO plus CT vs. CT plus placebo (or CT alone in Checkmate 649) in patients with advanced GC with intestinal subtype showed OS benefit with IO + CT (HR = 0.78; 95% CI, 0.69–0.87; p < 0.0001) with no observed heterogeneity (I2 = 0%). (B) Pooled analysis of four randomized phase III trials assessing IO plus CT vs. CT plus placebo (or CT alone in Checkmate 649) in patients with advanced GC with diffuse subtype showed significant OS benefit for IO + CT (HR = 0.87; 95% CI, 0.74–1.01; p = 0.0067) with moderate heterogeneity (I2 = 39%). Individual study estimates are represented by blue squares (size proportional to study weight), and the pooled effect is shown as a red diamond. Values < 1.0 indicate improved survival with IO plus CT. CI, confidence interval; CT, chemotherapy; HR, hazard ratio; IO, PD-(L)1 inhibitor; SE, standard error. [file Supplementaryfile1.jpg]

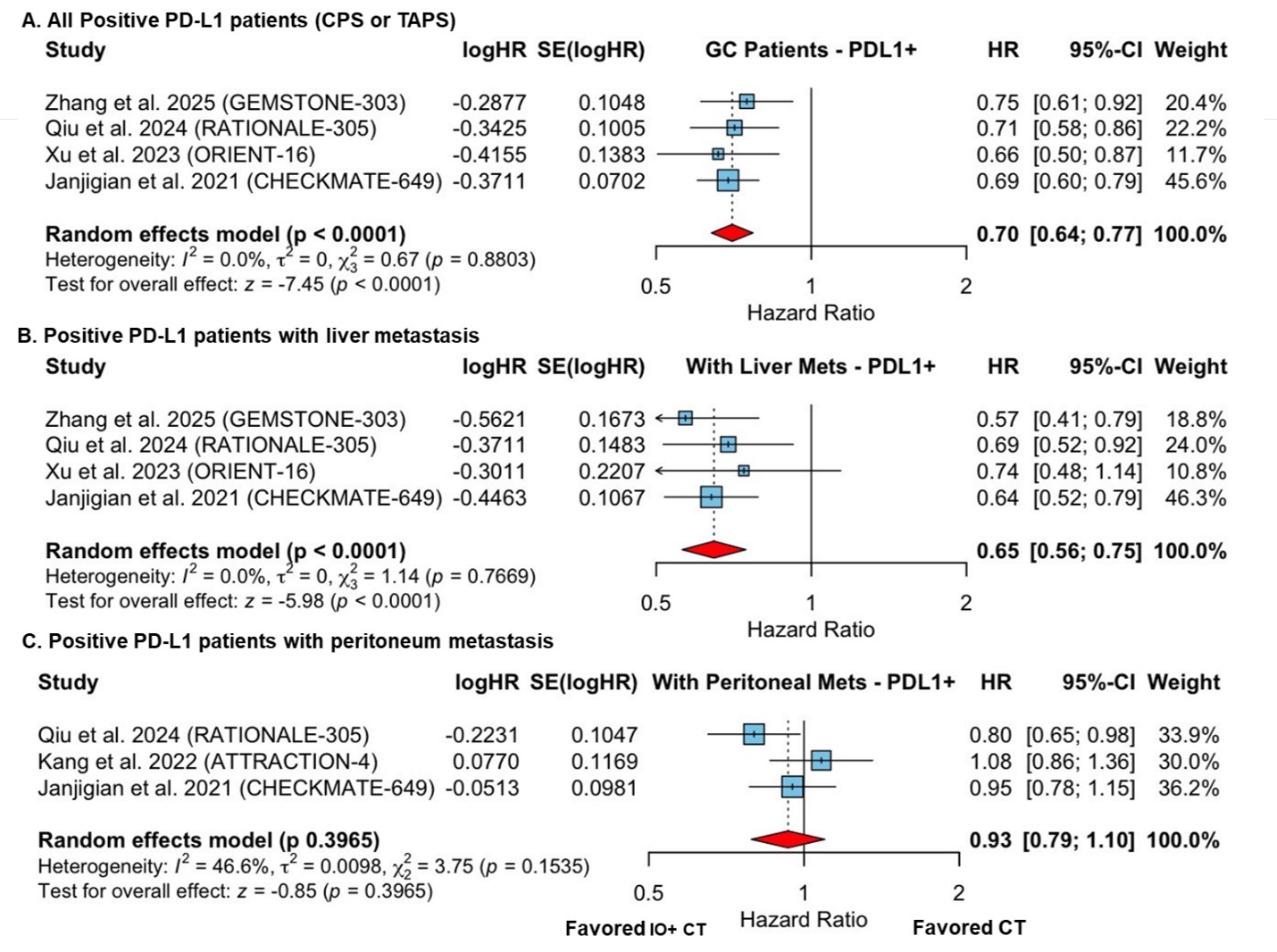

Supplement: Supplementary Figure S2 — OS benefit of first-line chemo-immunotherapy in PD-L1–positive gastric and gastroesophageal junction cancer, stratified by metastatic site. Forest plots depict HRs for OS comparing IO plus CT versus CT alone across randomized phase III trials. (A) In the overall PD-L1–positive population, chemo-immunotherapy was associated with a significant survival benefit (pooled HR 0.70, 95% CI 0.64–0.77; p < 0.0001) with no detectable heterogeneity. (B) Among PD-L1–positive patients with LM, a consistent and pronounced OS benefit was observed (pooled HR 0.65, 95% CI 0.56–0.75; p < 0.0001). (C) No significant survival benefit was demonstrated in PD-L1–positive patients with PM (pooled HR 0.93, 95% CI 0.79–1.10; p = 0.40), with moderate between-study heterogeneity. OS, overall survival; CT, chemotherapy; HR, hazard ratio; IO, PD-(L)1 inhibitor; PM, Peritoneum metastasis; LM, liver metastases; SE, standard error. [file Supplementaryfile2.jpg]
